# Is Intraoperative Ultrasound a Valuable Tool for Brain Arteriovenous Malformation Diagnosis and Treatment? A Case Report

**DOI:** 10.7759/cureus.5888

**Published:** 2019-10-11

**Authors:** Raphael Bertani, Karl R Abi-Aad, Caio Perret, Ahmad K AlMekkawi, Ruy Monteiro

**Affiliations:** 1 Neurosurgery, Hospital Municipal Miguel Couto, Rio de Janeiro, BRA; 2 Neurosurgery, Mayo Clinic, Phoenix, USA; 3 Neurosurgery, Federal University of the State of Rio De Janeiro, Rio de Janeiro, BRA; 4 Neurosurgery, Brigham and Women's Hospital, Brookline, USA

**Keywords:** brain avm, arteriovenous malformations (avms), intraoperative ultrasonography, intracerebral hemorrhage (ich), neurosurgery, cerebrovascular neurosurgery, emergency medicine

## Abstract

The localization of arteriovenous malformations (AVMs) intraoperatively in the setting of an acute intracerebral hemorrhage (ICH) is crucial to avoid damage of delicate vascular structures that may even further exacerbate the bleed. Currently, surgical mapping using preoperative angiographic is the standard of practice. We report the use of intraoperative ultrasound for the diagnosis and localization of an AVM in the case of a 61-year-old female with reported iodine contrast allergy and previous severe reaction, in a setting with limited resources, without other imaging options or timely transfer to another facility readily available. Immediate surgical care was warranted to avoid further deterioration of the patient; intraoperative diagnosis and localization of the suspected underlying lesion were done using ultrasound. The ultrasound display showed tubular anechoic intertwined structures that demonstrated bidirectional flow, which is suggestive of an AVM. The intraoperative diagnosis allowed the surgeon to avoid an inadvertent approach to the vascular malformation nidus or vessels, which could have further complicated the case. We believe that intraoperative ultrasound may be valuable for the neurosurgeons today in many settings. Despite the fact that this case occurred in a scenario with limited resources and no other imaging method (such as magnetic resonance imaging (MRI), magnetic resonance angiography (MRA)) available, we advise readers not to rely solely on intraoperative ultrasound.

## Introduction

Brain arteriovenous malformations (AVMs) are generally sporadic congenital developmental vascular lesions that occur in about 0.1% of the population, usually as single lesions [1-3]. AVM rupture is a rare cause of hemorrhagic stroke corresponding to around 2% of all intracranial hemorrhages [4]. The annual rate of bleeding is around 2.2%, doubling to 4.5% if the AVM has been previously ruptured [4].

They typically present in patients from 10 to 40 years with intracerebral hemorrhage (ICH) (41%-79%) and/or seizures (11%-33%), depending on the location, size, and venous drainage [1,5-8]. These lesions may be identified on cross-sectional imaging, such as computed tomography (CT) and magnetic resonance imaging (MRI). Contrasted imaging such as CT-angiography (CTA) provides more sensitivity than conventional CT and MRI. Furthermore, MRI-angiography (MRA) is even more sensitive than CTA, especially for the characterization of vascular features required for surgical planning. Although CT and MRI-angiography have been increasingly used, conventional angiography remains the gold standard and is routinely used for diagnosis and surgical planning [9-11].

## Case presentation

A 61-year-old woman, previously asymptomatic, was admitted to the emergency department of our hospital, with a history of a sudden headache and right-sided weakness. Family members reported that her symptoms first appeared in the morning and were progressively worsening. Upon examination, the patient was awake, disoriented, with a Glasgow Coma Scale (GCS) score of 14 (motor response: 6; verbal response: 4; eye-opening: 4). She was hemodynamically stable (blood pressure: 136/68 mm Hg, heart rate: 93 beats per minute, oxygen saturation: 99%) with right-sided hemiparesis (power grade 3).

**Figure 1:**
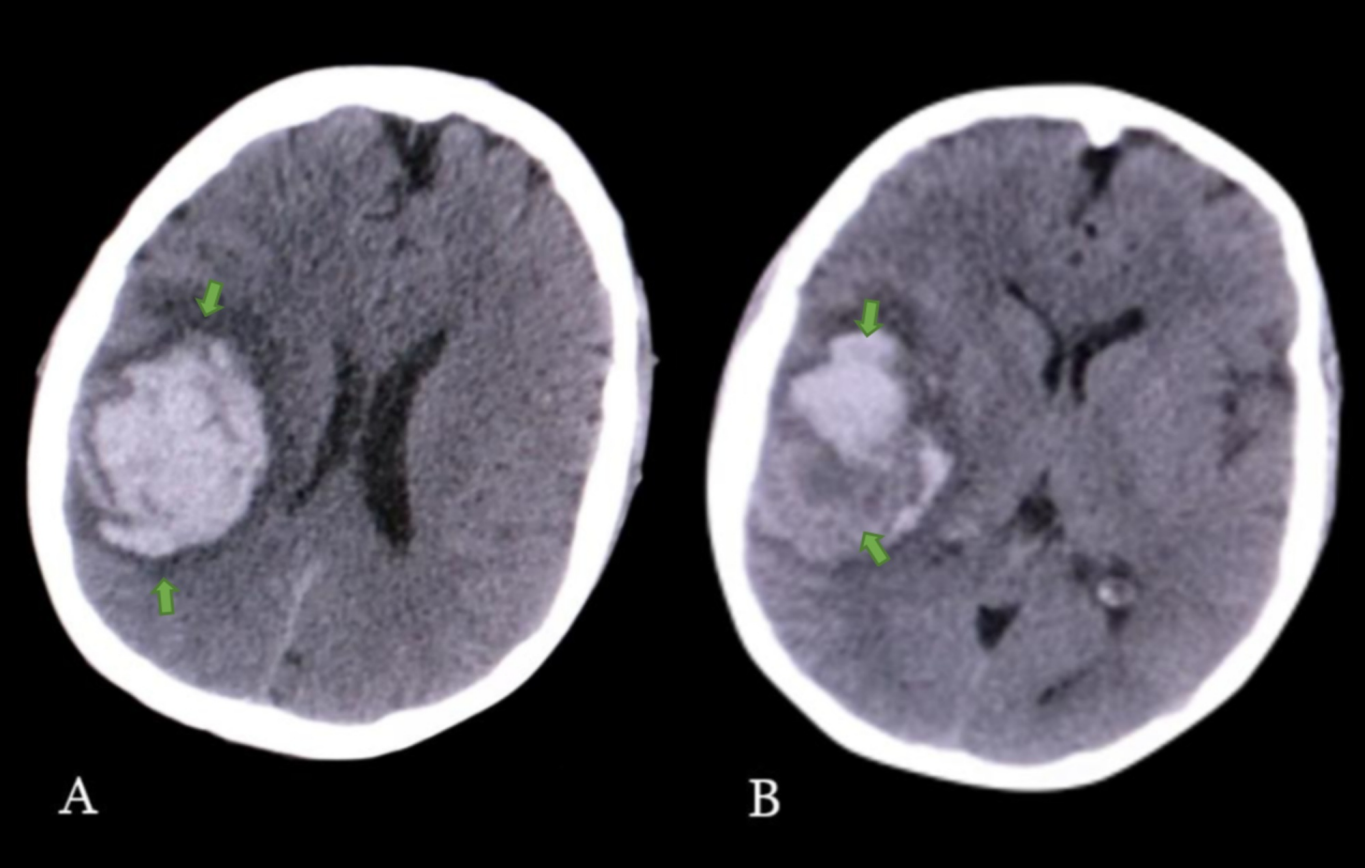
Axial section of the computed tomography (CT) scan A: Predominantly hyperdense, right frontoparietal mass-occupying lesion, suggestive of hemorrhage with midline shift; B: Heteregenous densities (hypodense and hyperdense) within the same lesion. Volume estimation of 47 mL^3^.

CT scan showed a 47 mL intraparenchymal frontal hematoma with midline shift (Figure 1). The hematoma was mostly hyperdense but heterogeneous, with hypodense areas within.

**Figure 2:**
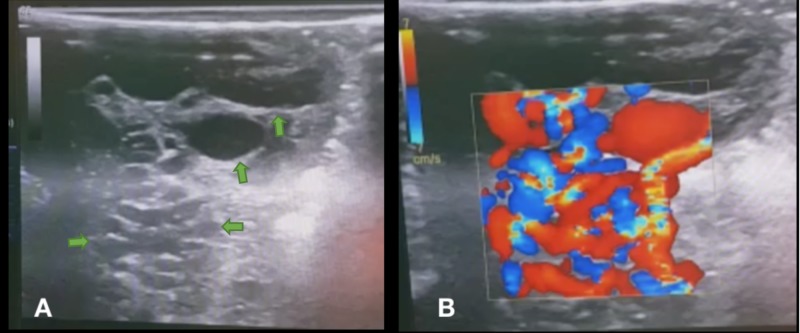
Intraoperative ultrasound A: Gray-scale ultrasound with linear probe showing multiple hypoechoic/anechoic tubular structures (arrows) within the brain parenchyma. B: Doppler function showing bidirectional, high-blood flow within the tubular structures, suggestive of arteriovenous malformation (AVM) vessels.

This peculiar aspect raised suspicion of a possible underlying brain vascular malformation, and a CT-angiography was suggested. Upon explaining the case to family members, and taking the history of the patient, an allergy to intravenous iodine contrast with severe reaction was reported, thus rendering the team unable to immediately confirm the suspected diagnosis. The patient’s neurological state declined in the meantime to GCS 12 (motor response: 6; verbal response: 3; eye-opening: 3), with surgery becoming imperative. At the moment, there was limited resource availability and an unknown delay in transferring the patient to another facility. With the family’s consent, the patient was taken to the operating room for partial drainage of the hematoma. After the opening of the dura-mater, an ultrasound linear-array probe was used to successfully identify multiple, abnormal vascular structures and the presence of blood flow with the doppler function (Figure 2) (Video 1).

**Video 1:** Intraoperative ultrasound Intraoperative ultrasound with linear probe at the brain parenchyma over the intraparenchymal hemorrhage. To the right: Gray-scale ultrasound showing multiple hypoechoic/anechoic tubular structures within the brain parenchyma. To the left: Doppler function showing bidirectional, high-blood flow within the tubular structures, suggestive of blood-flow within an AVM.

**Figure 3:**
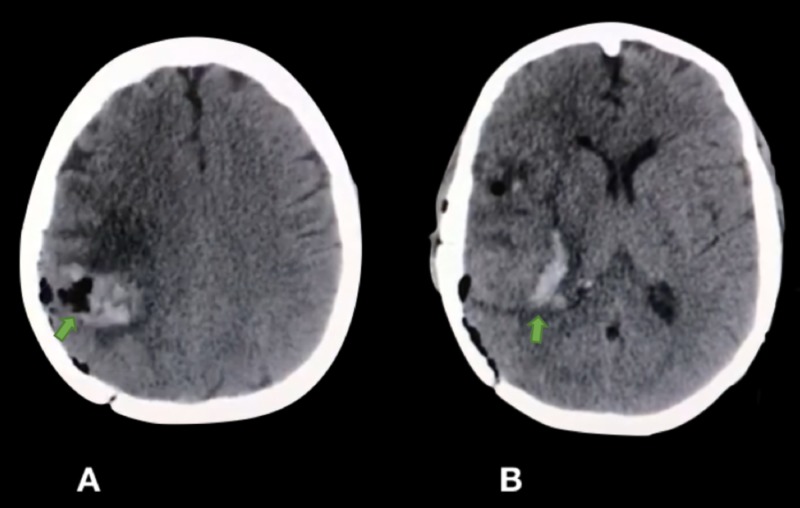
Axial sections of the post-operative computed tomography (CT) scan A: Partial drainage of the hematoma and pneumoencephalon within the lesion; B: Partial drainage of the hematoma.

This enabled the team to perform safer, partial drainage of the hematoma, in an attempt to relieve intracranial hypertension without causing catastrophic blood loss by accidental vascular injury (Figure 3). 

The patient was taken to the intensive care unit (ICU) postoperatively. She recovered to being oriented, scoring 15 points on the GCS and partially recovering power on the right side to grade 4 and was transferred to another center for endovascular treatment of the AVM.

## Discussion

Ultrasound is a safe and reliable tool that may be used intraoperatively for brain AVMs, with its use also being reported for uterine and liver AVMs. It allows the neurosurgeon to gain further insight into the anatomy and blood flow parameters of brain AVMs that had been, in the most part, previously studied and diagnosed with other imaging methods (CT, MRI, angiography, and variations) and also to ensure complete resection [12-17].

Although angiography remains gold-standard with CTA or MRA as second-choices for diagnosis and study of AVMs, in this particular scenario, these standard imaging methods for AVM diagnosis were either unavailable or contraindicated for this specific patient. Intraoperative ultrasound proved to be a valuable tool to support the diagnostic hypothesis and an important visual aid to improve safety during surgery, enabling more precise locations of the structures the surgeon desires to avoid, such as the vessels and the nidus of the AVM.

Another option for this patient would have been performing a decompressive hemicraniectomy (DC), completely avoiding the hematoma and the underlying malformation. Although we have experience with this procedure and it has been reported in the literature regarding ICH, there are no standardized criteria for performing DC in the setting of ICH [18-19]. In our department, we opt for DC for ICH cases with: 1. GCS of 8 or less, 2. Estimated hematoma volume ≥ 25mL^3^ which will not be evacuated (unable to approach safely) 3. Obliteration of basal cisterns 4. Significant midline shift (≥ 5mm with associated brain swelling)

In this case, although the estimated volume of the hematoma exceeded 25mL^3^, the patient presented a deterioration of the GCS from 14 to 12 and did not present a significant midline shift or obliteration of basal cisterns on CT scan. Moreover, during the intraoperative evaluation, the hematoma was deemed to be safely evacuated using microsurgical techniques and tools with delicate, soft suction and continuous irrigation.

The use of intraoperative ultrasound may also be of particular importance in the setting of ICH since angiography and MRI may not reveal the underlying malformation in the acute setting [20].

## Conclusions

In our case, the standard imaging methods (angiography, CTA, MRA) for AVM diagnosis were either not readily available or contraindicated for this specific patient due to contrast medium allergy. We conclude that, although angiography remains gold-standard and other imaging methods should be used as second choice (CTA, MRA, MRI), in an emergency setting where a complete vascular study is not made possible and surgical evacuation of the hematoma was required, intraoperative ultrasound was a safe and valuable diagnostic tool that improved safety during surgery and enabled localization of the delicate vascular structures, such as the nidus and malformed vessels pertaining to the arteriovenous malformation, that were to be avoided, since accidental damage could have been catastrophic. A safe partial hematoma evacuation was made possible without any injury to the nidus or vessels of the AVM, that could be visualized with intraoperative ultrasound. The patient was safely and timely transferred to another unit for endovascular treatment. We believe intraoperative ultrasound should we added to neurosurgeon's armamentarium and neurosurgical training as it can be used in a myriad of different cases and situations and more studies should be conducted to assess other possibilities of its use.
